# Do sleep bruxism patients experience higher complication or failure rates with monolithic lithium disilicate or zirconia molar crowns?

**DOI:** 10.1038/s41432-026-01230-2

**Published:** 2026-06-20

**Authors:** Afrida Khalid, Thikriat Al-Jewair

**Affiliations:** Department of Orthodontics, University at Buffalo School of Dental Medicine, Buffalo, NY USA

**Keywords:** Dentistry, Enamel

## Abstract

**A Commentary on:**

**Bomicke W, Schmitter M, Waldecker M, et al**.

Ceramic crowns and sleep bruxism: 3-year results of a randomized controlled trial. J Dent 2026;170:106691. https://doi.org/10.1016/j.jdent.2026.106691.

**Design:**

Prospective randomized controlled clinical trial with a 3-year follow-up evaluating the influence of Sleep Bruxism (SB) on the clinical performance of monolithic ceramic molar crowns.

**Case Selection:**

A total of 109 patients requiring a single molar crown were enrolled. SB diagnosis included a structured questionnaire, clinical examination, and portable electromyography device (BruxOFF). Participants were allocated into four groups: lithium disilicate with SB, lithium disilicate without SB, zirconia with SB, and zirconia without SB. Standardized crown preparation, fabrication, and cementation protocols were used. Outcomes assessed were technical complications, survival, and success rates. The study time points were 1 week, 6 months, and 1, 2, and 3 years.

**Data analysis:**

Survival was defined as restoration remaining in situ without replacement. Success is defined as restoration without biological or technical complications. Fisher’s exact test was used to compare outcomes between SB and non-SB groups, with statistical significance set at α= 0.05.

**Results:**

No technical complications occurred during the 3-year observation period. Survival rates remained high across all groups (95.2–100%), while success rates ranged from 81.5% to 95.8% for both ceramic materials. No statistically significant differences were observed between SB and non-SB patients or between lithium disilicate and zirconia crowns.

**Conclusions:**

Monolithic lithium disilicate and zirconia molar crowns demonstrated favorable clinical performance over three years, and SB was not associated with increased failure or complication risk.

## GRADE Rating:



## Commentary

The study^1^ demonstrated generally good adherence to CONSORT reporting standards, particularly regarding trial design, participant flow, intervention description, and outcome reporting. However, limitations in assessor blinding, sample size, and protocol transparency reduce the overall quality of evidence.

According to the Revised Cochrane Risk of Bias Tool (RoB2), the study was graded overall to have “some concerns” risk of bias rather than high risk. Although the randomization and allocation methods were appropriate and an intention-to-treat approach was used, concerns remained regarding baseline imbalances between groups, moderate attrition, and the potential for detection bias because treating clinicians performed a substantial proportion of the follow-up assessments on their own restorations (41%). Additionally, the relatively small sample size and absence of technical complications limited the statistical power to detect rare failures.

SB is a parafunctional activity characterized by rhythmic or non-rhythmic masticatory muscle activity during sleep^2^. Although not classified as a disorder, SB has been associated with several clinical consequences, including tooth wear, temporomandibular disorders, headaches, and muscular pain. The etiology of SB is multifactorial and may involve central nervous system activity, psychological stress, anatomical factors, environmental influences, and genetic predisposition. Importantly, SB has also been linked with obstructive sleep apnea (OSA), with some theories suggesting that SB may function as a compensatory mechanism to help maintain airway patency during sleep. Effective management of SB often requires a multidisciplinary approach involving sleep physicians, dentists, and other healthcare professionals^3^.

Sleep bruxism has traditionally been regarded as a major risk factor for ceramic restoration failure, frequently leading clinicians to avoid all-ceramic crowns in these patients. This randomized controlled trial challenges this long-standing clinical assumption by demonstrating comparable survival and complication rates between bruxism and non-bruxism patients restored with monolithic ceramic crowns. The findings suggest that SB alone may not increase complication or failure rates over a 3-year period.

A notable strength of this study lies in its structured bruxism assessment, which incorporated electromyographic monitoring in addition to clinical indicators and patient reporting—allowing SB to be identified through subject, clinical, and device-based assessment methods^2^. Many previous studies relied solely on indirect signs such as tooth wear, potentially overestimating the relationship between bruxism and restoration failure. By improving diagnostic rigor, the authors provide stronger evidence regarding the true clinical impact of SB.

The absence of technical complications observed during follow-up suggests that zirconia and lithium disilicate crowns may tolerate functional loading associated with SB more effectively than previously assumed. The results reflect the improved fracture resistance and durability of contemporary monolithic ceramic materials compared with earlier veneered restorations. The high crystalline content of zirconia and the lithium disilicate crystal structure likely contribute to their enhanced mechanical strength, crack resistance, and ability to withstand occlusal stresses.

Despite these strengths, several limitations should still be considered before broadly accepting these findings. The sample size remained relatively moderate and may not detect all possible or some uncommon complications or failures. Additionally, some of the crown follow-up examinations were performed by the original clinicians, introducing potential detection bias when assessing the longevity and success of the crown. The study also followed up only after three years, which is a relatively short follow-up period for evaluating long-term ceramic performance. The absence of polysomnography, the current gold standard, should also be acknowledged.

Conflict-of-interest considerations appear minimal, as the authors reported no competing interests and funding was provided through an independent research grant. Nonetheless, clinicians should recognize that the absence of complications over three years does not eliminate long-term risk.

Overall, this study provides clinically relevant evidence suggesting that SB alone should not contraindicate monolithic ceramic crowns. Instead, treatment planning should prioritize biomechanical principles, preparation design, and patient-specific risk factors.

Practice points

- Sleep bruxism alone should not be considered a contraindication for monolithic lithium disilicate or zirconia molar crowns, as medium-term clinical outcomes appear comparable to non-bruxism patients.
- Long-term studies are still required to evaluate fatigue-related complications in bruxism patients.
